# Supplementary material for: A novel reporter allele for monitoring Dll4 expression within the embryonic and adult mouse
Source: Biol Open. 2018 Feb 8;7(3):bio026799. doi: 10.1242/bio.026799 (PMC5898260; doi:10.1242/bio.026799)
Supplement: Supplementary information [file biolopen-7-026799-s1.pdf]

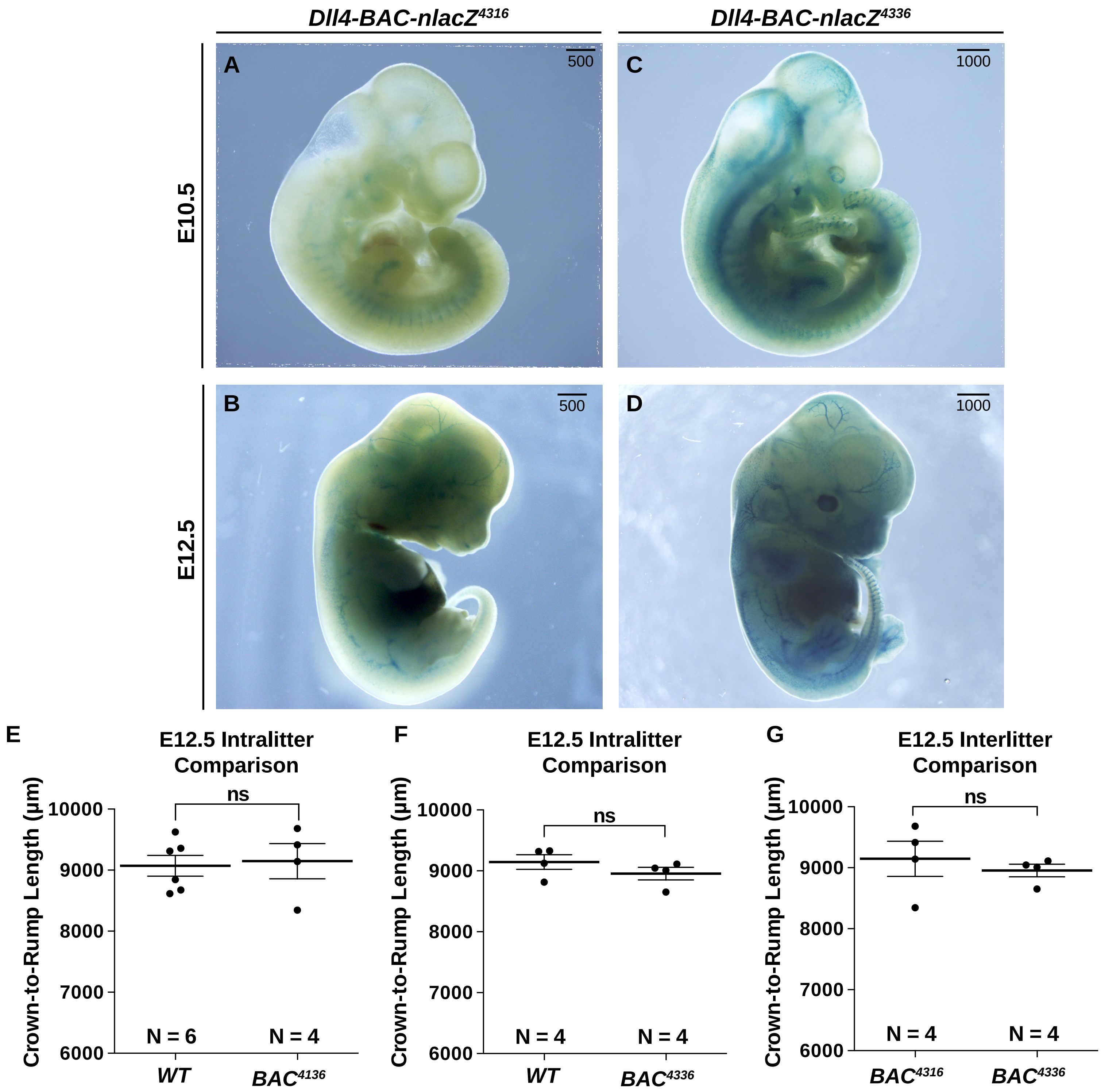

H

Genotype	*Dll4*$^{LacZ/+}$	*Dll4-BAC-nLacZ*^{4336}	*Dll4-BAC-nLacZ*^{4316}
**Predicted LacZ+**	50%	50%	50%
**Observed LacZ+**	35.76%	48%	45%
**Total (n)**	165	128	58

Figure S1: Viability assessment and comparative reporter expression between *Dll4-BAC-nLacZ*^{4316} and *Dll4-BAC-nLacZ*^{4336}.

A-B, Representative wholemount images of stained E10.5 (A) or E12.5 (B) *Dll4-BAC-nLacZ*^{4316} embryos. **C-D,** Representative wholemount images of stained E10.5 (C) or E12.5 (D) *Dll4-BAC-nLacZ*^{4336} embryos. **E-G**, Body measurements at E12.5 between transgenic and non-transgenic 4316 littermates (E), 4336 littermates (F), or 4316 and 4336 transgenic animals (non-littermates) (G). **H,** Viability assessment between *Dll4*$^{lacZ/+}$ and both *Dll4-BAC-nLacZ* founder lines. All crosses were between heterozygous *Dll4* reporter animals and WT animals with an expected yield of 50% lacZ-positive offspring. Data is presented as an average ± S.E.M. Comparisons were made by Student's T-Test. Scale bars depicted are in μm.

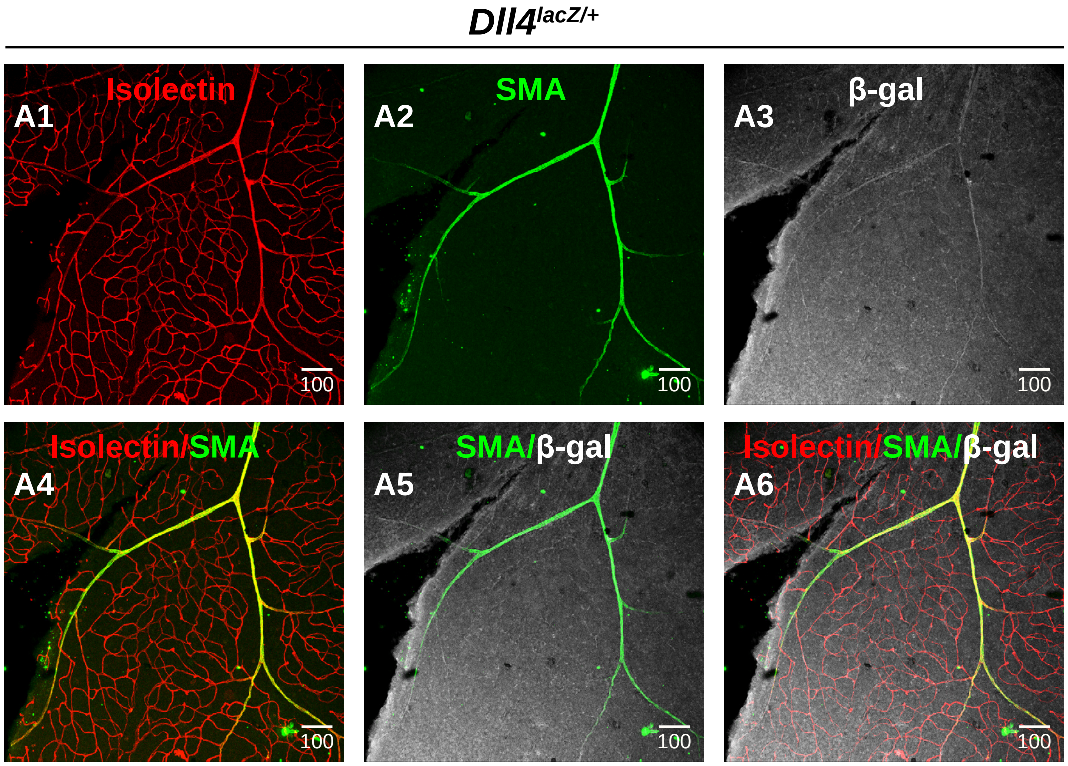

Figure S2: Comparative *Dll4* expression in postnatal and adult retinas.

A1-A6, Immunohistochemistry and indirect immunofluorescent detection of isolectin B4 (A1), smooth muscle actin (SMA) (A2), β-gal (A3), and merged (A4-A6) images from representative *Dll4*$^{lacZ/+}$ adult retinas. Scale bars depicted are in μm.

Biology Open • Supplementary information
